# Construction of g-C_3_N_4_ and FeWO_4_ Z-scheme photocatalyst: effect of contact ways on the photocatalytic performance†

**DOI:** 10.1039/c8ra02882f

**Published:** 2018-05-21

**Authors:** Cong Wang, Guanlong Wang, Xiufang Zhang, Xiaoli Dong, Chun Ma, Xinxin Zhang, Hongchao Ma, Mang Xue

**Affiliations:** School of Light Industry and Chemical Engineering, Dalian Polytechnic University Dalian China 116034 zhangxf010807@163.com +86 411 86323736 +86 411 86323508

## Abstract

Photocatalysis has been regarded as an attractive strategy for the elimination of contaminants, but its performance is usually limited by the fast recombination of photogenerated electron–holes. A heterojunction photocatalyst could achieve the effective separation of electron–holes. However, the electrons migrate to the less negative band while holes move to the less positive band, leading to a weakened redox ability. Z-scheme photocatalysis is a feasible way to realize the efficient separation of photogenerated electron–holes without sacrificing the reductive ability of electrons and oxidative ability of holes. In this work, a new Z-scheme photocatalyst, composed of g-C_3_N_4_ (photocatalyst I), FeWO_4_ (photocatalyst II) and RGO (electron mediator), was fabricated through a facile hydrothermal and mixing method. The effect of contact ways (the electron mediator firstly combined with photocatalyst I or with photocatalyst II) on the Z-scheme photocatalytic performance was investigated. The photocatalytic removal rate of rhodamine B (RhB) was largely enhanced by the construction of a Z-scheme photocatalyst, compared with the g-C_3_N_4_/FeWO_4_ composite without RGO. The contact ways could affect the photocatalytic ability of a Z-scheme photocatalyst. The enhanced photocatalytic performance was attributed to the Z-scheme induced efficient separation of photogenerated charge carriers. Furthermore, remaining holes (on the VB of FeWO_4_) or remaining electrons (on the CB of g-C_3_N_4_) with powerful oxidation or reduction ability would promote the photocatalytic degradation of RhB.

## Abstract 2

g-C_3_N_4_ (photocatalyst I), FeWO_4_ (photocatalyst II) and RGO (electron media) constitutes a novel Z-scheme photocatalyst for effective RhB removal.

## Graphical abstract

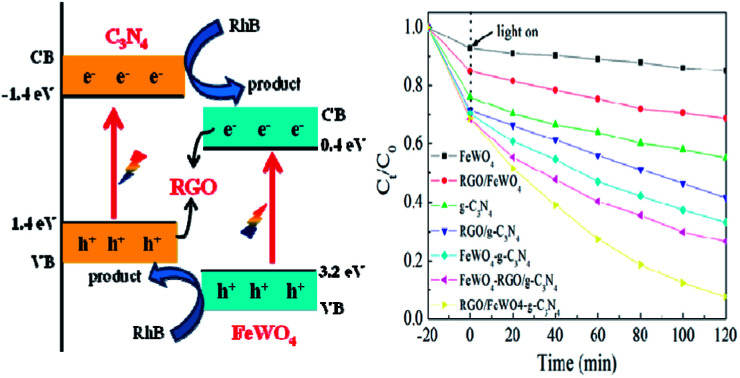

## 1. Introduction

Over the past few decades, semiconductor photocatalysis has been widely applied in the destruction of organic pollutants in air or water.^1,2^ Various semiconductor materials, such as oxides,^3^ sulfides,^4^ nitrides,^5^ or solid solutions,^6^ have been exploited as single-phase photocatalysts. However, rapid recombination of the photoexcited electron–hole pairs usually occurs in single-phase photocatalysis, which will restrict the photocatalytic activity. To address this issue, composite photocatalysts are commonly used to make efficient charge carrier separation.^7–10^ Nevertheless, photogenerated electrons in a composite photocatalysts migrate to and accumulate in less negative conduction bands, while holes migrate to and accumulate in less positive valence bands. Thus, from the point of view of thermodynamics, the oxidation performance of holes or the reduction ability of electrons is weakened compared with single-component photocatalysts. The Z-scheme photocatalytic system, which proceeds through a two-step photoexcitation (PS II of a photocatalytic oxidation system and PS I of a photocatalytic reduction system), is deemed to be an ideal approach to overcome this drawback.^11–13^ The Z-scheme photocatalytic system employs two photocatalysts (photocatalyst I for PS I and photocatalyst II for PS II) and a suitable electron transfer mediator. Photogenerated electrons within PS II combine with holes within PS I *via* the electron mediator. Thus, electrons with a high reduction ability in PS I and holes with a high oxidation ability in PS II are preserved and participate in the subsequent surface reaction. This unique advantage of powerful redox ability makes the Z-scheme system superior for water splitting and pollutant degradation. However, very limited progress in the construction of a Z-scheme system has been achieved, as it is difficult to control the desirable combination of holes and electrons *via* an electron mediator. The key point is that intimate interaction between the two photocatalysts must occur to allow the charge transfer. Up to now, some Z-scheme photocatalytic systems have been constructed for water splitting or pollutant decomposition.^14–19^ However, the effect of contact ways (the electron mediator firstly combined with photocatalyst I or with photocatalyst II) on the Z-scheme photocatalytic performance has not been thoroughly explored.

It has been proved that bulk g-C_3_N_4_, as a metal-free photocatalyst with a band gap of about 2.8 eV, can eliminate organic pollutants in water and split water under visible light irradiation.^20,21^ In 2009, Wang *et al.* first reported the use of g-C_3_N_4_ for hydrogen or oxygen production from water splitting under visible light irradiation.^22,23^ It is usually synthesized *via* a series of heating treatments from a simple precursor such as low-cost melamine.^24^ The CB and VB levels of g-C_3_N_4_ are −1.4 eV and 1.4 eV *vs.* NHE, respectively.^25^ So it is a promising candidate for photocatalyst I due to its relatively negative conduction band level. FeWO_4_ is also a visible-light-driven photocatalyst, which can decompose some pollutants *via* photocatalysis. In previous work, Kovacs *et al.* first prepared FeWO_4_ through a general hydrothermal method.^26^ Gao *et al.* also reported the synthesis of FeWO_4_ microplates in a general low-temperature hydrothermal route.^27^ The prepared three-dimensional FeWO_4_ materials displayed excellent photocatalytic activity. It is chosen as photocatalyst II because of its relatively positive valence band potential (3.2 eV *vs.* NHE). Furthermore, the band potential matching is another point to be considered for the construction of a Z-scheme photocatalytic system. That is, the conduction band potential of photocatalyst II should be more negative than that of the valence band of photocatalyst I. The conduction band potential of FeWO_4_ is 0.4 eV *vs.* NHE, which is more negative than that of the valence band of g-C_3_N_4_. RGO, a solid electron transfer medium for a Z-scheme photocatalytic system, is commonly employed for the combination of photogenerated carriers.^28^ It is known that RGO possesses good electron transfer ability and a large surface area.^29,30^ RGO could form tight contacts with two semiconductors and form an electron transfer bridge for Z-scheme induced charge recombination.

In this study, a new Z-scheme photocatalyst, with g-C_3_N_4_ as photocatalyst I, FeWO_4_ as photocatalyst II and RGO as electron mediator was fabricated for enhanced photocatalytic ability. RGO/g-C_3_N_4_–FeWO_4_ (RGO firstly combined with g-C_3_N_4_) and RGO/FeWO_4_–g-C_3_N_4_ (RGO firstly combined with FeWO_4_) were prepared to investigate the effect of the contact ways of the two photocatalysts and the electron mediator on the Z-scheme photocatalytic performance. The photocatalytic ability was evaluated by degradation of rhodamine B (RhB) under visible light.

## 2. Experimental

### 2.1 Preparation of photocatalysts

GO was synthesized from natural graphite flakes by a modified Hummers' method.^31^ GO was dispersed in water by ultrasonication and the GO concentration was 2 mg mL^−1^.

The g-C_3_N_4_ was synthesized by calcination. A certain amount of melamine was put into an alumina crucible which was first heated at 550 °C for 4 h with a temperature rise rate of 10 °C min^−1^. To obtain thin g-C_3_N_4_, the above powder was further heated at 550 °C for 4 h with a temperature rise rate of 5 °C min^−1^. The resultant g-C_3_N_4_ was collected for further use.

RGO/FeWO_4_ was synthesized by a hydrothermal method. In the process, 0.811 g of FeCl_3_·6H_2_O (3 mmol) and 0.990 g of Na_2_WO_4_·2H_2_O (3 mmol) were dissolved in 36 mL of deionized water. The solution was kept stirring for 2 h. Then, the prepared GO suspension (14 mL) was dropped into the solution, and the mixture was put into an ultrasonic vibration generator for 30 min. The above mixture was then poured into a 50 mL Teflon-sealed autoclave and heated to 180 °C for 8 h. After that, the precipitate was rinsed with distilled water and alcohol and collected by centrifugation. The above precipitate was dried in an oven at 120 °C for 12 h. In addition, RGO or FeWO_4_ were fabricated by the same method without an Fe or W source or GO to serve as a control.

RGO/g-C_3_N_4_ was fabricated by a hydrothermal process. In detail, the ultra-thin g-C_3_N_4_ (95 mg) was dispersed in 45 mL of deionized water by ultrasonication, and then a GO suspension (5 mL) was dispersed in the solution. The pH value of the above mixture was adjusted to 3.5. The above mixture was then added into a 50 mL Teflon-sealed autoclave and heated to 180 °C for 8 h. After that, the precipitate was rinsed with distilled water and alcohol and collected by centrifugation. The obtained precipitate was dried in an oven at 80 °C for 12 h.

FeWO_4_/g-C_3_N_4_ was synthesized by a hydrothermal method. In the process, 0.811 g of FeCl_3_·6H_2_O (3 mmol) and 0.990 g of Na_2_WO_4_·2H_2_O (3 mmol) were dissolved in 36 mL of deionized water. The solution was kept stirring for 2 h. Then, the prepared g-C_3_N_4_ suspension was added into the solution, and the mixture was put into an ultrasonic vibration generator for 30 min. The above mixture was then poured into a 50 mL Teflon-sealed autoclave and heated to 180 °C for 8 h. After that, the precipitate was rinsed with distilled water and alcohol and collected by centrifugation. The above precipitate was dried in an oven at 120 °C for 12 h.

The Z-scheme photocatalyst, RGO/FeWO_4_–g-C_3_N_4_ was prepared by the following process, g-C_3_N_4_ (100 mg) and RGO/FeWO_4_ (100 mg) were suspended in 50 mL of water, and then the mixture was stirred for 2 h. The precipitate was washed and dried for 5 h under the condition of 80 °C. To prepare composites with identical structures (the photocatalyst combined firstly with RGO will perhaps occupy the “surface active sites” of RGO), FeWO_4_-RGO/g-C_3_N_4_ (FeWO_4_ and RGO/g-C_3_N_4_) were prepared by the same process. And also, FeWO_4_–g-C_3_N_4_ and RGO-FeWO_4_/g-C_3_N_4_ (RGO and FeWO_4_/g-C_3_N_4_) were prepared by the same process as a reference.

### 2.2 Characterization

The crystal type of a sample was investigated by XRD (Rigaku, Japan) with Cu Kα radiation, an accelerating voltage of 40 kV, and current of 30 mA. A DRS spectrum was recorded using a UV-visible diffuse reflector (UV-2450, Shimadzu, Japan), and the wavelength range was from 200 to 800 nm. The specific surface areas (BET) of the powders were analyzed by the nitrogen adsorption–desorption method using a Micromeritics ASAP 2020 nitrogen adsorption apparatus (USA) at 77 K. PL spectra were obtained on a Fluorescence Spectrometer (LS 55, PerkinElmer, America). The morphology of the photocatalysts was observed using an optical microscope (LEICA; DM 2500M). The surface chemical bonds of the photocatalysts were investigated by XPS. The elemental composition of the synthesized materials was examined by Energy Dispersive X-ray Spectroscopy (EDX).

### 2.3 Measurement of the photocatalytic activity

Photocatalytic activity was evaluated by the degradation of RhB under visible light irradiation. A 300 W Xe lamp was employed as the light source, and the light was passed through a glass filter which could shield any light with a wavelength below 400 nm. Experiments were carried out as follows: the photocatalyst (0.08 g) was suspended in RhB solution (5 mg L^−1^, 80 mL). Before illumination, the suspension was stirred for 40 min in the dark to ensure the establishment of an adsorption–desorption equilibrium. A specified volume of suspension (5 mL) was collected every 20 min, and the photocatalyst was removed by centrifugation (9500 rpm, 10 min). Then, the residual RhB concentration was calculated by the absorbance of the solution at 554 nm, measured by a UV-vis spectrophotometer (Shimadzu, UV-2450). To clarify the possible oxidizing species in the photocatalytic system, EDTA, *p*-benzoquinone (BQ), and *tert*-butyl alcohol (*t*-BuOH) were employed as scavengers for holes, O_2_˙^−^, and ·OH, respectively. The concentration of the scavenger was 1 mM.

## 3. Results and discussion

### 3.1 XRD characterization

**Fig. 1:**
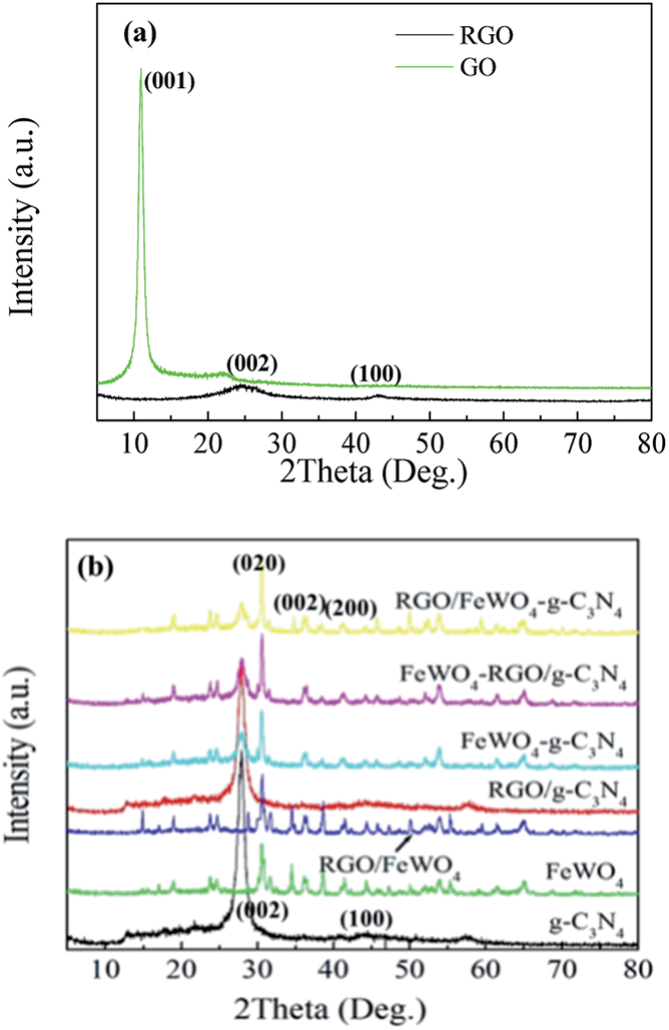
XRD patterns of (a) GO and RGO, and (b) g-C_3_N_4_, FeWO_4_, RGO/FeWO_4_, RGO/g-C_3_N_4_, FeWO_4_–g-C_3_N_4_, FeWO_4_-RGO/g-C_3_N_4_ and RGO/FeWO_4_–g-C_3_N_4_.

To determine the phase structure, XRD patterns were recorded. Fig. 1(a) shows the XRD patterns of GO and RGO, in which a peak was found at 9.9° in the GO pattern, which corresponds to (001) (*d* = 0.90 nm),^32^ and no obvious peak was identified in the RGO pattern, indicating that the regular stacking of GO has been destroyed during the hydrothermal process. Fig. 1(b) shows the XRD patterns of g-C_3_N_4_, FeWO_4_, RGO/g-C_3_N_4_, RGO/FeWO_4_, FeWO_4_–g-C_3_N_4_, RGO/FeWO_4_–g-C_3_N_4_ and FeWO_4_-RGO/g-C_3_N_4_. The XRD pattern of g-C_3_N_4_ exhibited a weak peak at 13.01° (*d* = 0.681 nm) and a strong peak at 27.5° (*d* = 0.326 nm), which can be indexed to the (002) and (100) diffraction planes of the graphite-like carbon nitride. The (002) diffraction and (100) diffraction relate to the characteristic interlayer stacking structure and the interplanar structural packing. The weak (100) diffraction indicated that the obtained g-C_3_N_4_ was thin,^33^ which could be attributed to the repeated exfoliation by calcination. The main reflection peaks in the FeWO_4_ pattern could be indexed to a monoclinic structure, according to the standard card (JCPDS-PDF, no. 27-0256).^34^ When compositing RGO, there were no new peaks in the pattern of RGO/g-C_3_N_4_ and RGO/FeWO_4_, indicating that the introduction of RGO could not change the phase structure of g-C_3_N_4_ or FeWO_4_. A similar conclusion could be drawn based on there being no new founding peaks in the patterns of the other three compounds (FeWO_4_–g-C_3_N_4_, FeWO_4_-RGO/g-C_3_N_4_ and RGO/FeWO_4_-g-C_3_N_4_) compared with the original materials (FeWO_4_ and g-C_3_N_4_, FeWO_4_ and RGO/g-C_3_N_4_, RGO/FeWO_4_ and g-C_3_N_4_). According to Scherrer's equation: *D* = *k*λ/*β* cos *θ*, where *D* is the average crystal diameter, *k* is a constant of value 0.89, *λ* is the radiation wavelength of 0.154 nm, *β* is the full width at half maximum of the diffraction peak, and *θ* is the Bragg angle, the crystal size of RGO/FeWO_4_–g-C_3_N_4_ was 45.33 nm. Moreover, the main peaks of the original materials were all observed in the patterns of the compounds, showing that Z-scheme photocatalysts had been successfully prepared.

### 3.2 Microscope images

**Fig. 2:**
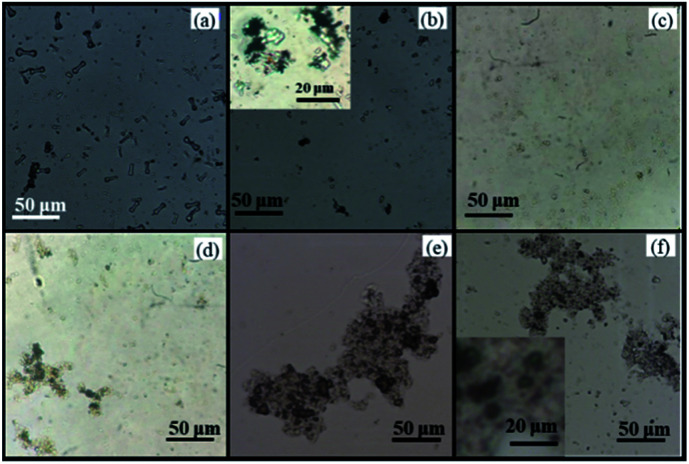
Optical microscope images of FeWO_4_ (a), RGO/FeWO_4_ (b), g-C_3_N_4_ (c), RGO/g-C_3_N_4_ (d), FeWO_4_-RGO/g-C_3_N_4_ (e) and RGO/FeWO_4_–g-C_3_N_4_ (f).

Fig. 2 displays the optical microscope images. In Fig. 2(a), FeWO_4_ shows a bone-like shape, and its size was 20–30 μm in length and 5 μm in width. In Fig. 2(b), some small black points were found. RGO loading on FeWO_4_ made the particle image darker. Compared with single FeWO_4_, the particle size of FeWO_4_ in RGO/FeWO_4_ (below 10 μm) was obviously reduced. The exact reason was not clear, but it can be speculated that RGO was a model which restricted the growth of FeWO_4_ particles. As shown in Fig. 2(c), g-C_3_N_4_ was flaky. The image becomes lighter, which could be attributed to good light transmission by thin C_3_N_4_. A darker image was found in Fig. 2(d), indicating that RGO and g-C_3_N_4_ were successfully hybridized. In Fig. 2(e), it was found that RGO/g-C_3_N_4_ was successfully and uniformly loaded onto FeWO_4_ particles. From Fig. 2(f), g-C_3_N_4_ was successfully loaded onto RGO/FeWO_4_ particles, and maintained a uniformity of distribution, which could promote charge transfer between the materials. The elemental composition of the different synthesized materials was examined by EDX. As shown in Table S1,† RGO/FeWO_4_–g-C_3_N_4_ and FeWO_4_-RGO/g-C_3_N_4_ are composed of C, N, O, Fe and W. Compared with the weight ratio of C/N in g-C_3_N_4_ (0.64), the weight ratios of C/N in RGO/FeWO_4_–g-C_3_N_4_ (0.76) and FeWO_4_-RGO/g-C_3_N_4_ (0.81) are higher. This means that RGO has been successfully introduced.

### 3.3 BET

The specific surface area is an important parameter for photocatalysts, because photocatalysis mainly occurs on and near the surface of photocatalysts. The specific surface area was determined by N_2_ adsorption/desorption, and the calculated values are displayed in Table 1. It can be seen from Table 1 that the specific surface area of FeWO_4_ was 24.8 m^2^ g^−1^. After coupling with RGO, the BET value of FeWO_4_/RGO increased (38.4 m^2^ g^−1^), which could be ascribed to the large specific surface area of RGO and the deceased particle size of FeWO_4_. Due to its thin layer structure, g-C_3_N_4_ possessed a large surface area (86.7 m^2^ g^−1^). Compared with that of g-C_3_N_4_, the specific surface area of RGO/g-C_3_N_4_ (83.9 m^2^ g^−1^) did not obviously change because of the similar specific surface areas of RGO and g-C_3_N_4_. Table 1 also shows that RGO/FeWO_4_–g-C_3_N_4_ and FeWO_4_-RGO/g-C_3_N_4_ had similar specific surface areas.

**Table 1:** Specific surface areas of g-C_3_N_4_, FeWO_4_, RGO/g-C_3_N_4_, RGO/FeWO_4_, FeWO_4_–g-C_3_N_4_, RGO/FeWO_4_–g-C_3_N_4_ and FeWO_4_-RGO/g-C_3_N_4_ from N_2_ adsorption/desorption

Sample	RGO	FeWO_4_	RGO/FeWO_4_	g-C_3_N_4_	RGO/g-C_3_N_4_	FeWO_4_–g-C_3_N_4_	FeWO_4_-RGO/g-C_3_N_4_	RGO/FeWO_4_–g-C_3_N_4_
BET (m^2^ g^−1^)	178.5	24.8	38.4	86.7	83.9	75.6	75.3	75.3

### 3.4 XPS

**Fig. 3:**
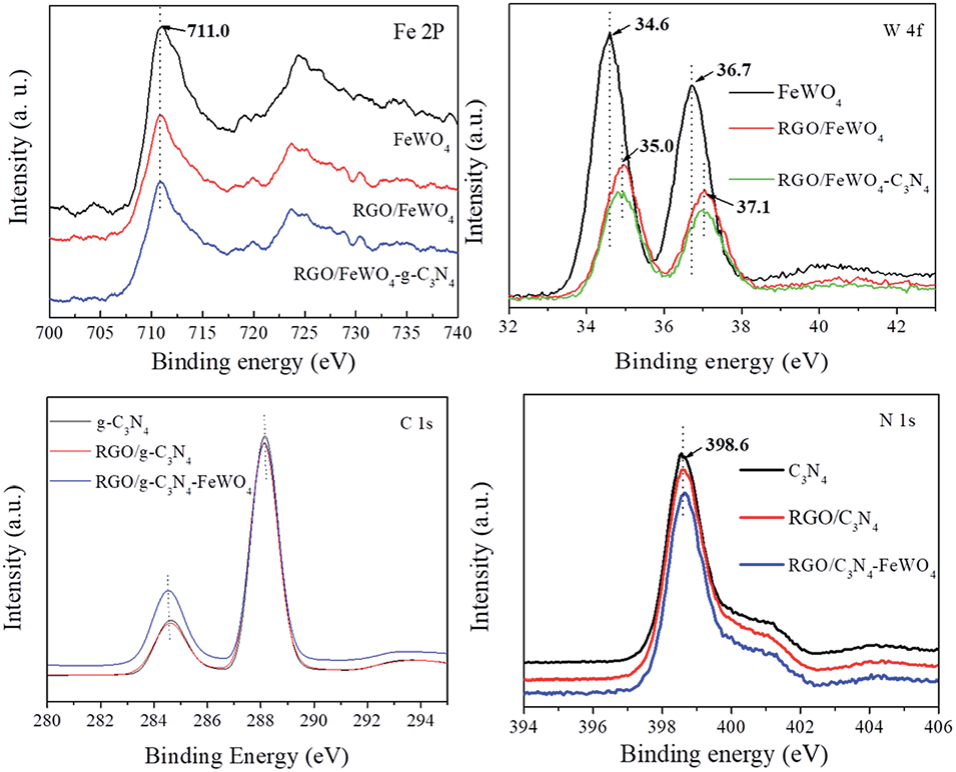
XPS spectra of Fe 2P, W 4f, C 1s and N 1s.

To discover the surface chemical bonds of the photocatalysts, XPS was recorded and the results are shown in Fig. 3. The Fe 2p spectrum of FeWO_4_ exhibited one peak (710.0 eV), and no obvious shift in peaks was found in the spectra of RGO/FeWO_4_ or RGO/FeWO_4_–C_3_N_4_. However, in the W 4f spectra, higher binding energies of RGO/FeWO_4_ and RGO/FeWO_4_–C_3_N_4_ (35.0 eV and 37.1 eV) were determined compared with those of FeWO_4_ (34.6 and 36.7 eV). The XPS results first revealed that the chemical environment of W was changed after RGO coupling, which could be attributed to the generation of chemical bonds between RGO and FeWO_4_ during the hydrothermal process. A similar result was also found in other literature.^34^ Furthermore, no new chemical bonds were generated during C_3_N_4_ loading onto RGO/FeWO_4_ by physical mixing. The peak found in the C 1s spectrum of C_3_N_4_ centered at 284.6 eV was attributed to the C–C bond of contaminated carbon. The peak at 288.0 eV corresponds to the tertiary carbon C–N_3_.^35^ There was no obvious shift in the peaks of RGO/C_3_N_4_ and RGO/C_3_N_4_–FeWO_4_, indicating there was no new generation of chemical bonds between RGO and C_3_N_4_ in RGO/C_3_N_4_ or RGO/C_3_N_4_–FeWO_4_.

### 3.5 DRS

**Fig. 4:**
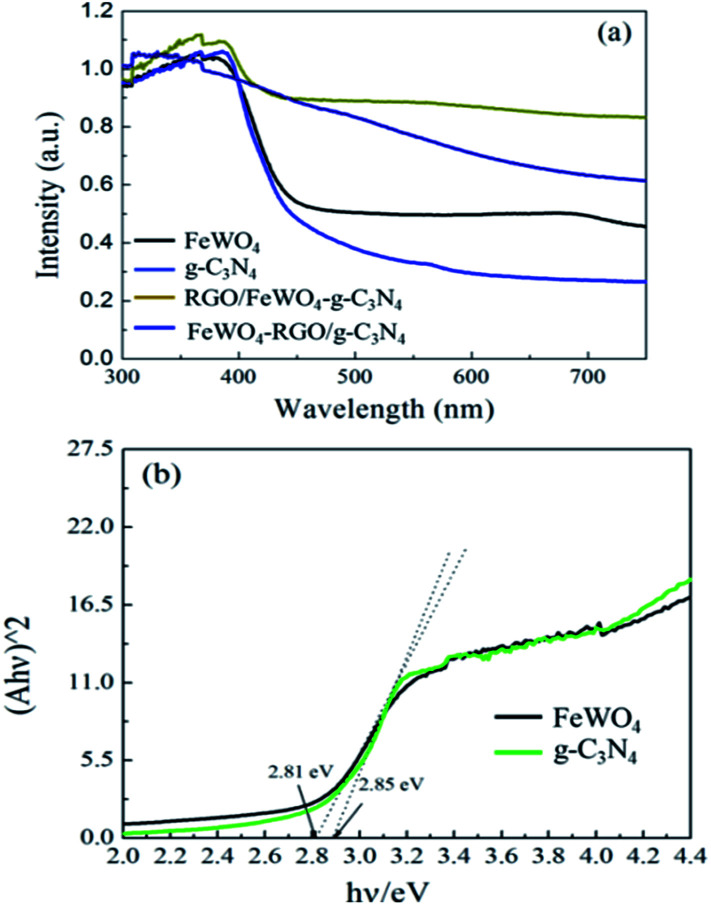
(a) DRS and (b) band gap calculation by the Kubelka–Munk function of FeWO_4_, g-C_3_N_4_, FeWO_4_-RGO/g-C_3_N_4_ and RGO/FeWO_4_–g-C_3_N_4_.

DRS and band gap calculations are illustrated in Fig. 4. Both samples of g-C_3_N_4_ and FeWO_4_ exhibited significant absorption in the visible light region. The band gaps calculated by the Kubelka–Munk function of g-C_3_N_4_ and FeWO_4_ were 2.85 and 2.81 eV, respectively, which were similar to the reported values.^27,29^ RGO/FeWO_4_–g-C_3_N_4_ and FeWO_4_-RGO/g-C_3_N_4_ showed enhanced absorption intensity compared with C_3_N_4_ and FeWO_4_, especially in the range of 400-800 nm. It was speculated that the dark color of the powders made them absorb more photons.

### 3.6 PL spectra

**Fig. 5:**
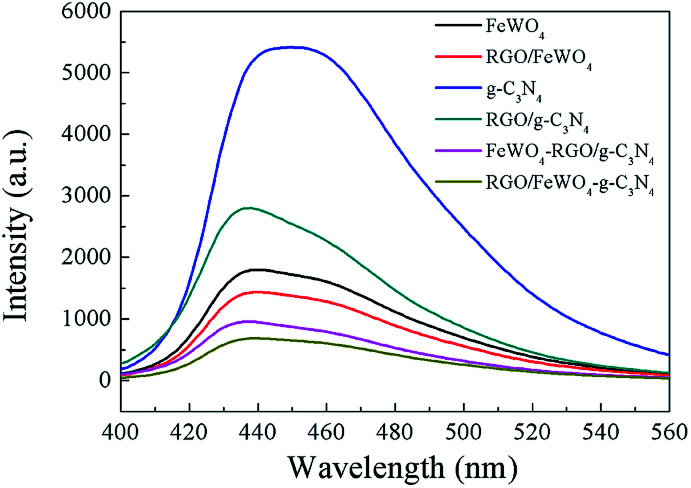
PL spectra of FeWO_4_, RGO/FeWO_4_, g-C_3_N_4_, RGO/g-C_3_N_4_, g-C_3_N_4_–FeWO_4_, FeWO_4_-RGO/g-C_3_N_4_ and RGO/FeWO_4_–g-C_3_N_4_.

PL spectra analysis was used to discover the separation efficiency of photogenerated charge carriers in semiconductors. A larger PL intensity means more recombination of photogenerated charge pairs. In the detected PL spectra (Fig. 5), the intensity of RGO/g-C_3_N_4_–FeWO_4_ was lower than that of RGO/g-C_3_N_4_, and the intensity of RGO/g-C_3_N_4_ was smaller than that of g-C_3_N_4_. The result indicated that loading RGO onto g-C_3_N_4_ could improve the separation performance of photogenerated charge carriers, caused by the migration of photogenerated electrons from g-C_3_N_4_ to RGO. The lowest PL intensity of RGO/g-C_3_N_4_–FeWO_4_ indicated that the separation of photogenerated charge carriers could be improved through the construction of a Z-scheme system. A similar conclusion could be reached by comparing the PL intensity with FeWO_4_, RGO/FeWO_4_ and RGO/FeWO_4_–g-C_3_N_4_, which further confirmed the enhanced separation of photogenerated charge carriers in the Z-scheme photocatalyst. With respect to a comparison of RGO/g-C_3_N_4_–FeWO_4_ and RGO/FeWO_4_–g-C_3_N_4_, the PL intensity of RGO/FeWO_4_–g-C_3_N_4_ was slightly lower, indicating that the separation of photogenerated charge carriers on it was more effective. Since less recombination of photogenerated charge carriers leads to better photocatalytic efficiency, it could be expected that the Z-scheme photocatalyst would have high photocatalytic ability, and RGO/FeWO_4_–g-C_3_N_4_ would have the highest photocatalytic performance.

### 3.7 Photocatalytic activity

**Fig. 6:**
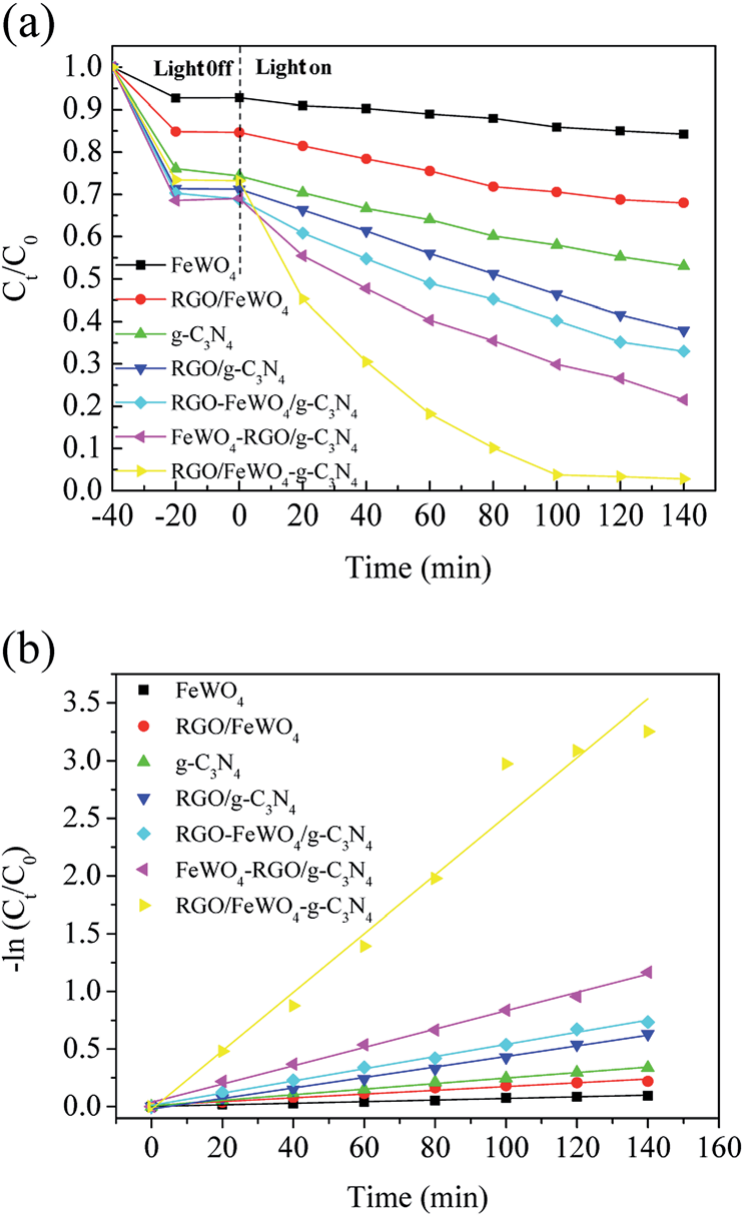
(a) *C*_*t*_/*C*_0_*versus* reaction time and (b) the kinetic curves for photocatalytic degradation of RhB on FeWO_4_, RGO/FeWO_4_, g-C_3_N_4_, RGO/g-C_3_N_4_, FeWO_4_–g-C_3_N_4_, FeWO_4_-RGO/g-C_3_N_4_ and RGO/FeWO_4_–g-C_3_N_4_ under visible light irradiation.

The photocatalytic performances of the photocatalysts were evaluated through RhB degradation under visible light irradiation, and the results are shown in Fig. 6. From Fig. 6(a), before light irradiation, only 7.4% of RhB was removed on FeWO_4_ after 20 min, meaning that the adsorption of RhB on FeWO_4_ was poor. After RGO loading, 23.9% of RhB with g-C_3_N_4_ was removed, which could be attributed to the large specific surface area of RGO. RhB removal on RGO/g-C_3_N_4_ was slightly improved compared with that on g-C_3_N_4_, which is possibly attributed to the small difference between the specific surface areas of g-C_3_N_4_ and RGO/g-C_3_N_4_. The RhB removals on FeWO_4_–g-C_3_N_4_, FeWO_4_-RGO/g-C_3_N_4_ and RGO/FeWO_4_–g-C_3_N_4_ were similar to that of RGO/g-C_3_N_4_. After visible light illumination for 120 min, about 15.0% of RhB was removed in the presence of FeWO_4_, and the removal on g-C_3_N_4_ was 31.2%, indicating that FeWO_4_ and g-C_3_N_4_ could be excited by visible light and could degrade RhB in a photocatalytic process. After RGO hybridisation, under the same reaction conditions, the removals of RhB on RGO/FeWO_4_ and RGO/g-C_3_N_4_ were 32.1% and 58.4%, respectively. The enhanced photocatalytic ability could be attributed to the improved separation of photogenerated charge carriers of RGO/FeWO_4_ and RGO/g-C_3_N_4_.

**Fig. 7:**
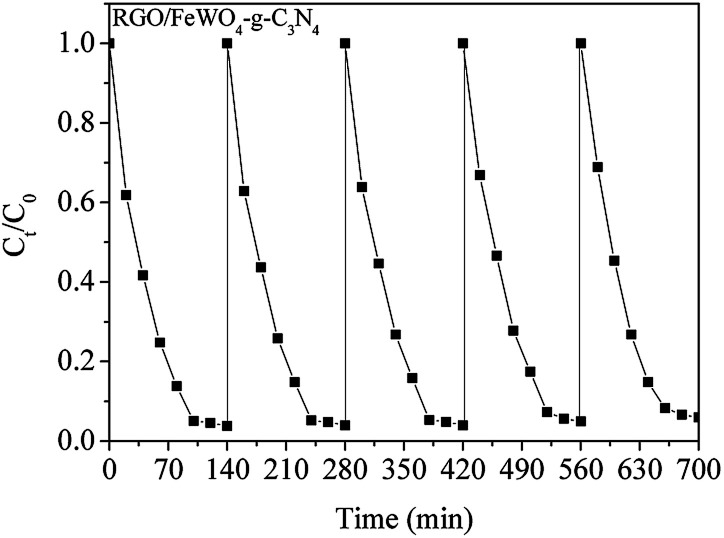
The stability of RGO/FeWO_4_–g-C_3_N_4_ for photocatalytic degradation of RhB under visible light illumination.

RhB removals by FeWO_4_-RGO/g-C_3_N_4_ and RGO/FeWO_4_–g-C_3_N_4_ were 73.5% and 92.3%, respectively, which were higher than those of previously mentioned photocatalysts, certifying that a Z-scheme structure could boost the photocatalytic efficiency. To further confirm the Z-scheme effect, the photocatalytic ability of FeWO_4_–g-C_3_N_4_ and RGO/FeWO_4_–g-C_3_N_4_, which had the same amounts of g-C_3_N_4,_ FeWO_4_ and RGO to those of FeWO_4_-RGO/g-C_3_N_4_ and RGO/FeWO_4_–g-C_3_N_4_ was evaluated as a reference. Under the same conditions, the RhB removal rates were significantly lower than those of FeWO_4_-RGO/g-C_3_N_4_ and RGO/FeWO_4_–g-C_3_N_4_, confirming that the enhanced photocatalytic performance of FeWO_4_-RGO/g-C_3_N_4_ and RGO/FeWO_4_–g-C_3_N_4_ was owing to the Z-scheme effect. The photocatalytic ability of RGO/FeWO_4_–g-C_3_N_4_ was higher than that of FeWO_4_-RGO/g-C_3_N_4_, suggesting that the contact ways (the electron mediator firstly combined with photocatalyst I or with photocatalyst II) could affect the Z-scheme photocatalytic performance. The photocatalytic process is fitted well with a pseudo-first-order reaction, and the kinetic curves of RhB degradation are depicted in Fig. 6(b). The kinetic constants of RhB degradation on FeWO_4,_ RGO/FeWO_4_, g-C_3_N_4_, RGO/g-C_3_N_4_, RGO-FeWO_4_/g-C_3_N_4_, FeWO_4_-RGO/g-C_3_N_4_ and RGO/FeWO_4_–g-C_3_N_4_ were calculated to be 0.00069, 0.0016, 0.0024, 0.0046, 0.0053, 0.0080 and 0.025 min^−1^, respectively. To elucidate the influence of RGO on the photocatalytic performance of RGO/FeWO_4_–g-C_3_N_4_, the RGO content in RGO/FeWO_4_–g-C_3_N_4_ was regulated. The results showed that RGO/FeWO_4_–g-C_3_N_4_ with the addition of 14 mg of RGO showed the best performance. The detailed information is displayed in Fig. S1.† The stability of g-C_3_N_4_-RGO/FeWO_4_ for the photocatalytic degradation of RhB was tested, as shown in Fig. 7. The photocatalytic activity of g-C_3_N_4_-RGO/FeWO_4_ shows almost no change after 5 recycling runs, and the RhB removal was maintained at above 90%. This result suggests that g-C_3_N_4_-RGO/FeWO_4_ possesses good stability and recyclability, which are significant for practical applications.

**Scheme 1:**
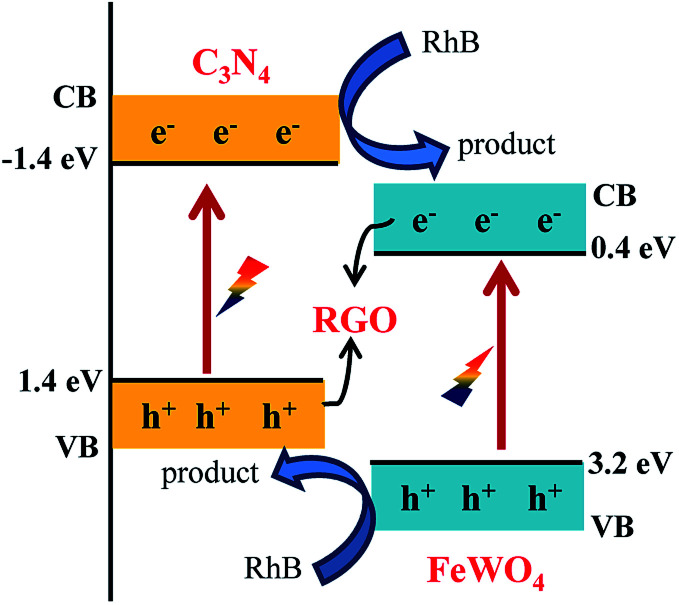
Proposed mechanism of enhanced photocatalytic ability of RGO/FeWO_4_–g-C_3_N_4_.

The reason for the improved photocatalytic performance was not exactly clear. It may be caused by the decreased FeWO_4_ particle size in RGO/FeWO_4_–g-C_3_N_4_, or the force deference of chemical bond (RGO and FeWO_4_ in RGO/FeWO_4_–g-C_3_N_4_) and physical contact (RGO and g-C_3_N_4_ in FeWO_4_-RGO/g-C_3_N_4_). A possible mechanism for RhB degradation with RGO/FeWO_4_–g-C_3_N_4_ as the photocatalyst was proposed and is shown in Scheme 1. When irradiated, the g-C_3_N_4_ and FeWO_4_ particles were excited and generated electrons and holes. Due to the match of the band positions, photogenerated electrons in FeWO_4_ would combine with holes in g-C_3_N_4_ through RGO (the electron mediator). The photogenerated holes in FeWO_4_ and photogenerated electrons in g-C_3_N_4_ were spatially separated. Thus, the separation of photogenerated holes and electrons was enhanced, and more electrons and holes would participate in the oxidation reaction. Meanwhile, remaining holes (on the VB of FeWO_4_) and electrons (on the CB of the g-C_3_N_4_) would promote the photocatalytic degradation of RhB because of its powerful oxidation ability and excellent reduction performance.

To further clarify the possible oxidizing species in the photocatalytic system, a radical quenching experiment was performed. In this experiment, EDTA, *p*-benzoquinone (BQ), and *tert*-butyl alcohol (*t*-BuOH) were employed as scavengers for holes, O_2_˙^−^, and ·OH, respectively. Fig. S2a† shows that the photocatalytic efficiency decreased after introducing EDTA and BQ, with the kinetic constant of RhB degradation decreasing from 0.025 min^−1^ to 0.020 min^−1^ and 0.017 min^−1^, respectively (Fig. S2b†), meaning the holes and O_2_˙^−^ participated in the RhB oxidation. When *t*-BuOH was added, the decrease in performance was more significant. The kinetic constant of RhB degradation decreased to 0.0084 min^−1^, suggesting that ·OH was the major oxidant in the photocatalytic system.

## 4. Conclusions

Z-scheme photocatalysts, in which g-C_3_N_4_, FeWO_4_ and RGO served as photocatalyst I, photocatalyst II and electron mediator, respectively, were successfully fabricated. The photocatalytic degradation rate of RhB was largely enhanced by the construction of a Z-scheme photocatalyst. The contact ways (the electron mediator firstly combined with photocatalyst I or with photocatalyst II) could influence the photocatalytic ability of the Z-scheme photocatalyst. The enhanced photocatalytic performance was attributed to the Z-scheme effect induced efficient separation of photogenerated electrons and holes. Furthermore, accumulated holes (on the VB of FeWO_4_) with powerful oxidation ability and remaining electrons (on the CB of the g-C_3_N_4_) with excellent reduction ability would promote the photocatalytic degradation of RhB. It was rationally confirmed that constructing a Z-scheme photocatalyst was a versatile method to explore a high performance photocatalyst, and the Z-scheme photocatalytic performance could be controlled by the contact ways.

## Conflicts of interest

There are no conflicts to declare.

## Supplementary Material

RA-008-C8RA02882F-s001
