# Supplementary material for: Construction of g-C3N4 and FeWO4 Z-scheme photocatalyst: effect of contact ways on the photocatalytic performance
Source: RSC Adv. 2018 May 21;8(33):18419–26. doi: 10.1039/c8ra02882f (PMC9080584; doi:10.1039/c8ra02882f)
Supplement: RA-008-C8RA02882F-s001 [file RA-008-C8RA02882F-s001.pdf]

Electronic Supplementary Material (ESI) for RSC Advances.
This journal is © The Royal Society of Chemistry 2018

Supporting Information for

Construction of g-C_3N_4 and $FeWO_4$ Z-scheme photocatalyst: Effect of contact ways on the photocatalytic performance

Cong Wang[1], Guanlong Wang[1], Xiufang Zhang*, Xiaoli Dong, Chun Ma, Xinxin Zhang, Hongchao Ma, Mang Xue

School of Light Industry and Chemical Engineering, Dalian Polytechnic University, Dalian, China, 116034.

*Corresponding authors. Tel.: +86 411 86323508; fax: +86 411 86323736.

E-mail address: zhangxf010807@163.com.

[1] These two authors contributed equally to this work

Table S1. The element composition of the materials (wt%).

Element	C	N	O	Fe	W
g-C_3N_4	64.00	36.00			
$FeWO_4$-RGO/g-C_3N_4	19.47	23.95	12.82	9.84	33.92
RGO/$FeWO_4$-g-C_3N_4	29.79	38.99	7.12	5.36	18.74

The influence of RGO content.

The RGO content in RGO/$FeWO_4$-g-C_3N_4 was varied by adding different mass of RGO (7 mg, 14 mg and 28 mg per 200 mg RGO/$FeWO_4$-g-C_3N_4) in the prepared process. As shown in the figure below, the RGO/$FeWO_4$-g-C_3N_4 with 14 mg RGO addition performed best. Based on the results, it could be speculated that the performance RGO/$FeWO_4$-g-C_3N_4 was enhanced with the increase of RGO, because the RGO is beneficial for electron transfer from CB of the $FeWO_4$ to the VB of the g-C_3N_4. However, the excess RGO would obstruct the light adsorption of $FeWO_4$, thus resulting in the performance decrease.

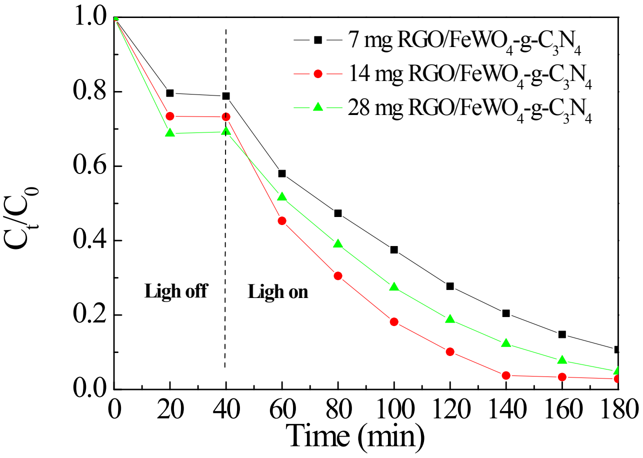

Fig S1. Photocatalytic degradation of RhB on RGO/$FeWO_4$-g-C_3N_4 with different RGO content under visible light.

Fig. S2. (a) Quenching effects of EDTA, BQ and t-BuOH on the photocatalytic RhB degradation. (b) the kinetic constant of RhB degradation in the presence of different scavengers.
